# DNA Methylation Signatures and the Contribution of Age-Associated Methylomic Drift to Carcinogenesis in Early-Onset Colorectal Cancer

**DOI:** 10.3390/cancers13112589

**Published:** 2021-05-25

**Authors:** Jihoon E. Joo, Mark Clendenning, Ee Ming Wong, Christophe Rosty, Khalid Mahmood, Peter Georgeson, Ingrid M. Winship, Susan G. Preston, Aung Ko Win, Pierre-Antoine Dugué, Harindra Jayasekara, Dallas English, Finlay A. Macrae, John L. Hopper, Mark A. Jenkins, Roger L. Milne, Graham G. Giles, Melissa C. Southey, Daniel D. Buchanan

**Affiliations:** 1 Colorectal Oncogenomics Group, Department of Clinical Pathology, The University of Melbourne, Parkville, Melbourne 3010, Australia; ji.joo@unimelb.edu.au (J.E.J.); mark.clendenning@unimelb.edu.au (M.C.); c.rosty@uq.edu.au (C.R.); khalid.mahmood@unimelb.edu.au (K.M.); peter.georgeson@unimelb.edu.au (P.G.); susan.preston@unimelb.edu.au (S.G.P.); harindra.jayasekara@cancervic.org.au (H.J.); 2 University of Melbourne Centre for Cancer Research, Victorian Comprehensive Cancer Centre, Parkville, Melbourne 3000, Australia; 3 Precision Medicine, Monash Health, Monash University, Clayton, Melbourne 3800, Australia; eeming.wong@monash.edu (E.M.W.); pierre-antoine.dugue@monash.edu (P.-A.D.); roger.milne@cancervic.org.au (R.L.M.); graham.giles@cancervic.org.au (G.G.G.); melissa.southey@monash.edu (M.C.S.); 4 School of Medicine, University of Queensland, Herston, Brisbane 4006, Australia; 5 Envoi Pathology, Brisbane 4059, Australia; 6 Melbourne Bioinformatics, The University of Melbourne, Parkville, Melbourne 3010, Australia; 7 Department of Medicine, The Royal Melbourne Hospital, University of Melbourne, Parkville, Melbourne 3050, Australia; ingrid.winship@mh.org.au (I.M.W.); finlay.macrae@mh.org.au (F.A.M.); 8 Genomic Medicine and Family Cancer Clinic, Royal Melbourne Hospital, Parkville, Melbourne 3050, Australia; 9 Centre for Epidemiology and Biostatistics, The University of Melbourne, Melbourne 3010, Australia; awin@unimelb.edu.au (A.K.W.); d.english@unimelb.edu.au (D.E.); j.hopper@unimelb.edu.au (J.L.H.); m.jenkins@unimelb.edu.au (M.A.J.); 10 Cancer Epidemiology Division, Cancer Council Victoria, Melbourne 3004, Australia; 11 Colorectal Medicine and Genetics, The Royal Melbourne Hospital, Parkville, Melbourne 3050, Australia; 12 Department of Clinical Pathology, The University of Melbourne, Parkville, Melbourne 3010, Australia

**Keywords:** early onset colorectal cancer, colorectal cancer, DNA methylation, age acceleration, epigenetic drift

## Abstract

**Simple Summary:**

The role of DNA methylation in the carcinogenesis of colorectal cancer (CRC) diagnosed <50 years of age (early-onset CRC or EOCRC) is currently unknown. In this study, we investigated the genome-wide DNA methylation of 97 tumour and 54 normal colonic mucosa samples from people with EOCRC with the aim of identifying unique DNA methylation signatures and determining the role of ageing-related DNA methylation drift and age-acceleration in EOCRC aetiology. We found extensive DNA methylation alterations associated with EOCRC carcinogenesis, including a unique signature comprising 234 loci compared with CRCs from people >50 years of age. CpGs that undergo ageing-related methylation drift were significantly altered in EOCRC, and accelerated ageing was also evident in normal mucosa from people with EOCRC. Our study is the first study to identify unique DNA methylation changes in EOCRC, contributing novel information that may aid future efforts towards EOCRC prevention.

**Abstract:**

We investigated aberrant DNA methylation (DNAm) changes and the contribution of ageing-associated methylomic drift and age acceleration to early-onset colorectal cancer (EOCRC) carcinogenesis. Genome-wide DNAm profiling using the Infinium HM450K on 97 EOCRC tumour and 54 normal colonic mucosa samples was compared with: (1) intermediate-onset CRC (IOCRC; diagnosed between 50–70 years; 343 tumour and 35 normal); and (2) late-onset CRC (LOCRC; >70 years; 318 tumour and 40 normal). CpGs associated with age-related methylation drift were identified using a public dataset of 231 normal mucosa samples from people without CRC. DNAm-age was estimated using epiTOC2. Common to all three age-of-onset groups, 88,385 (20% of all CpGs) CpGs were differentially methylated between tumour and normal mucosa. We identified 234 differentially methylated genes that were unique to the EOCRC group; 13 of these DMRs/genes were replicated in EOCRC compared with LOCRCs from TCGA. In normal mucosa from people without CRC, we identified 28,154 CpGs that undergo ageing-related DNAm drift, and of those, 65% were aberrantly methylated in EOCRC tumours. Based on the mitotic-based DNAm clock epiTOC2, we identified age acceleration in normal mucosa of people with EOCRC compared with normal mucosa from the IOCRC, LOCRC groups (*p* = 3.7 × 10^−16^) and young people without CRC (*p* = 5.8 × 10^−6^). EOCRC acquires unique DNAm alterations at 234 loci. CpGs associated with ageing-associated drift were widely affected in EOCRC without needing the decades-long accrual of DNAm drift as commonly seen in intermediate- and late-onset CRCs. Accelerated ageing in normal mucosa from people with EOCRC potentially underlies the earlier age of diagnosis in CRC carcinogenesis.

## 1. Introduction

Colorectal cancer (CRC) is the third most commonly diagnosed cancer and the second leading cause of cancer-related death worldwide [1]. CRC is a heterogeneous disease, for which increasing age is one of the strongest risk factors [1]. Screening programs have been implemented for people starting at age 50 years in many countries, which has contributed to a reduction in CRC incidence in this older age group [2]. In contrast, the incidence rate of CRC in people under 50 years of age has been increasing and now accounts for over 10% of all CRC diagnoses in the US [3]. Although monogenic cancer predisposition syndromes may explain up to 20% of all early-onset colorectal cancer (EOCRC) [4,5], it is unlikely to explain the increasing incidence of EOCRC. Currently, we do not know if EOCRC is molecularly distinct from later-onset CRC, a knowledge gap that hinders efforts to identify the cause/s of this increasing incidence.

CRCs acquire aberrant DNA methylation (DNAm), including focal hypermethylation leading to the silencing of tumour suppressor genes and global hypomethylation, resulting in genomic instability [6]. Global hypomethylation in tumour and hypermethylation in blood have been shown to be more frequent in EOCRC when compared with non-hereditary CRCs diagnosed at age >50 years [7,8]. To date, there has been no study exploring the aetiologic role of genome-wide DNA methylation changes in EOCRC. DNAm can be modified by environmental exposures, such as diet, and is likely to be a primary mechanism explaining environmentally-modulated EOCRC risk [9]. Further, DNAm changes at both cancer-related and ageing-related CpG loci have been identified in normal colonic mucosa (NM), suggesting that DNAm alterations may start very early in CRC tumourigenesis and predispose cells to neoplastic transformations [10,11,12]; hence they are a promising biomarker for predicting CRC risk [6].

Tissue-specific DNAm changes, including both hyper- and hypomethylation of CpGs, increase as part of the normal ageing process, a phenomenon referred to as “methylomic drift” [13]. The decades-long accumulation of methylomic drift is thought to underlie the association between ageing and CRC risk [13,14], where adverse endogenous and environmental stimuli can accelerate the rate of this methylomic drift [13,15]. Furthermore, DNAm is shown to be an accurate molecular indicator for biological age, and this has led to the development of numerous tools for estimating biological age using genome-wide DNAm data [16,17,18,19,20,21,22]. These tools have been used to investigate the difference between chronological and biological age, referred to as age acceleration (AA), where AA is associated with several diseases, including cancer risk [18].

Current evidence suggests that methylomic drift occurs more rapidly in colorectal neoplasia than in NM, but that cancer precursors frequently sojourn for decades before transitioning into cancer, implying that the founder premalignant cell typically arises early in life [14]. We hypothesise that EOCRC is characterised by specific DNAm aberrations, including those related to rapid methylomic drift and AA compared with later-onset CRC. In this study, we assessed genome-wide DNAm in tumour and NM samples from people with EOCRC (CRC diagnosed at age/AgeDx ≤ 50 years) as well as in people with intermediate-onset CRC (IOCRC; AgeDx between 50–70 years) and with late-onset CRC (LOCRC; AgeDx > 70 years). Our aims were to determine: (i) the molecular uniqueness of EOCRC tumours related to DNAm aberrations and the affected gene pathways and (ii) the role of methylomic drift and AA in NM and tumours from people with EOCRC compared with people with LOCRC. 

## 2. Materials and Methods

### 2.1. Study Participants

People affected with CRC were identified through the Australian Colorectal Cancer Family Registry (ACCFR) [23,24] and the Melbourne Collaborative Cohort Study (MCCS) [25]. The ACCFR recruited people diagnosed with CRC at <60 years of age identified by linkage to the Victorian Cancer Registry between 1998 and 2008. CRC-affected participants diagnosed at <50 years of age with available CRC tissue and matched normal colonic mucosa were selected for testing in this study. The MCCS is a prospective cohort study with 41,513 participants from the Melbourne metropolitan area with a mean age of 55 years when recruited between 1990 and 1994 [25]. By 31 December 2009, 1046 participants had a first histopathological diagnosis of invasive adenocarcinoma of the colon or rectum identified by a record linkage to the Victorian Cancer Registry following the baseline study visit. Germline and tumour characterisation of these CRC-affected probands from both studies has been previously described [26]. In total, the CRC tumours and matched NM samples (where available) from 769 individuals were included in the analysis. Individuals from both studies who carried a germline mutation in one or more of the DNA mismatch repair (MMR) genes (i.e., *MLH1*, *MSH2*, *MSH6*, and *PMS2*) or the *MUTYH* gene were excluded from the analysis. In addition, only CRCs that showed a normal and retained expression of all four MMR proteins by immunohistochemistry (i.e., MMR-proficient) were included in the analysis. For all subsequent analyses, samples were divided into three groups based on age at diagnosis (AgeDx): (1) Early or EOCRC (CRC AgeDx ≤ 50 years), (2) intermediate or IOCRC (AgeDx between 51 and 70 years), and (3) late-onset or LOCRC (AgeDx > 70 years).

### 2.2. DNA Extraction from FFPE Specimens

Formalin-fixed-paraffin-embedded (FFPE) tissue specimens from the tumours and matched NMs from the surgical resection margin were identified. All haematoxylin and eosin slides were reviewed by specialist GI pathologists, and the regions enriched in normal or tumour cells were marked up for macrodissection. DNA was extracted using the QIAamp DNA FFPE tissue kit (Qiagen, Hilden, Germany) and quantified using the Qubit dsDNA HS kit (Thermo Scientific, Carlsbad, CA, USA). Up to 500 ng of genomic DNA isolated from FFPE slides was bisulfite converted using the EZ DNA Methylation-Gold Kit (Zymo Research, Irvine, CA, USA), following the manufacturer’s instruction. The bisulfite-converted DNA was restored using the FFPE restore kit (Illumina, San Diego, CA, USA). The samples were processed in a research laboratory, and individual samples were subjected to multi-step quality control as previously described [27,28], to ensure adequate bisulfite conversion and DNA quality. To assess the reproducibility, technical replicates and positive controls were included in each batch of 96 samples.

### 2.3. DNA Methylation Array Processing and Bioinformatic Analyses

Genome-wide DNAm was profiled using the Infinium HumanMethylation450K array (HM450K; Illumina) and processed as previously described [27]. The staining and extension steps were performed using the TECAN EVO automated liquid handler (Männedorf, Switzerland). Raw data were imported into the R programming software and processed using the minfi Bioconductor package [29]. Raw intensity data were converted into MethylSet and normalised using Functional normalisation [30] with noob background correction [31], which are implemented in minfi [29]. Samples with mean detection *p*-values greater than 0.01 were removed from the analysis. Probes with a detection *p*-value greater than 0.05 were deemed noisy and excluded from the analysis. β (Beta) and M-values were calculated using the getBeta and getM function in minfi. β-values were used for presenting the data, and M-values were used for all statistical analyses as suggested previously [32]. To eliminate any sex-specific bias in DNAm, probes on sex chromosomes were removed from all analyses, and methylation levels were measured from 431,942 probes in total.

### 2.4. Statistical Analysis

Differentially methylated probes (DMPs) were identified using the limma Bioconductor package [33]. Differentially methylated regions (DMRs) were identified using the DMRcate Bioconductor package [34]. All *p*-values were FDR-adjusted for multiple testing unless stated otherwise. The differential methylation analyses between tumour and matched NMs (from the same individuals) were performed by utilising a pairwise design, therefore, intrinsically controlled for other covariates (e.g., sex, smoking). All plots were generated using ggplot2 R packages [35]. All statistical analyses were performed in R. KEGG and pathway analyses were performed on DMRs using the “gometh” function from the MissMethyl Bioconductor package [36].

### 2.5. Publicly Available DNA Methylation Dataset of Normal Colonic Mucosa and CRCs

CpGs associated with methylomic drift were identified using a publicly available methylation dataset of 231 NM samples collected from participants without CRC described as “healthy” from SMS and CICaRes cohorts (GEO Accession “GSE113904”) [14,37,38], which was downloaded using the minfi package [29]. One sample was removed due to missing anatomical site information. Raw “Drift-CpGs” were identified by performing a regression analysis between the M-values of individual CpG sites assessed by the HM450K and the age in years, which was fit using splines. The HM450K data of 47 EOCRC (AgeDx < 50) and 164 LOCRC (AgeDx ≥ 70) samples from TCGA were obtained using the “TCGAbiolinks” R package [39], after removing patients with Lynch syndrome and samples without relevant clinical information, including “age at diagnosis”. Raw iDAT files were downloaded and processed using *Minfi*. The data underwent Functional normalization with noob background correction [30].

### 2.6. DNA Methylation-Based Age Estimation

To calculate DNAm-based biological age (DNAmAge), we pooled the healthy NM data (*n* = 231) [14], with our NMs (*n* = 129) and CRC (*n* = 758) data. The pooled data underwent a ComBat batch correction [40] by treating individual slides (or “chips”) as a batch (Supplementary Figure S1). DNAmAge was assessed using five well-established algorithms. The Horvath clock was calculated via https://genetics.ucla.edu/new (accessed on 16 April 2020) [17]. The Hannum age [16] and PhenoAge [19] were estimated using the ENmix Bioconductor package [41]. The mitotic age was assessed by epiTOC (or “pcgtAge”) [21] and epiTOC2 (or “tnsc2”), which were calculated using the method and the R code detailed in Teschendorff et al. [20]. The average intrinsic rate of stem-cell division (irS) per year was also derived from the R code by inputting the corresponding samples’ chronological ages. Age acceleration (AA) was determined by estimating residuals from a linear regression of DNAmAge on chronological age and by adjusting for sex and cell heterogeneity (where relevant) as previously demonstrated [17,18,42,43]. For the Horvath clock, residual AA (“AgeAccelerationResidual”) was derived from the online calculator by uploading chronological age, gender, and tissue source [17].

## 3. Results

In total, we analysed genome-wide DNAm data from 758 CRC tumour and 129 histologically normal mucosa (NM) samples from 769 individuals stratified into EOCRC, IOCRC, and LOCRC subgroups (Table 1). Characteristics of the participants and their tumours are described in Table 1. A principal component analysis of all detected probes showed that samples clustered by tissue type, and no batch effect was observed (Supplementary Figure S2).

### 3.1. Differences in Genome-Wide DNA Methylation between Normal Mucosa and CRCs by Age-of-Diagnosis

**Figure 1:**
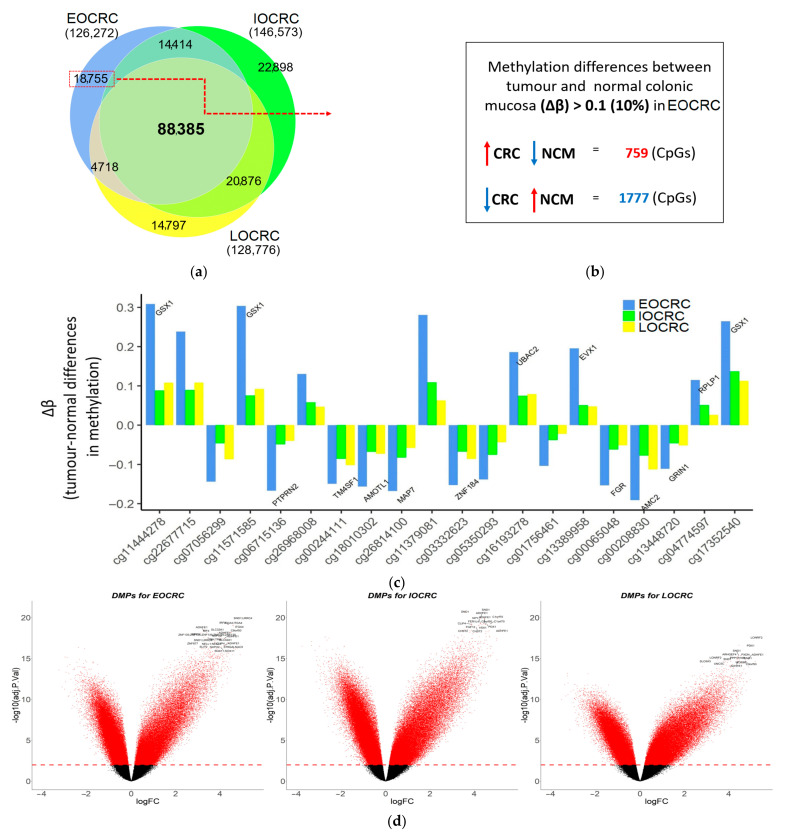
Differential methylation between matched tumour and normal pairs for three age groups. (**a**) (Proportional) Venn diagram showing the numbers of differentially methylated probes (DMPs) between tumour and matched normal mucosa samples for 41 EOCRCs, 34 IOCRCs, and 27 LOCRCs. (**b**) Of 18,755 DMPs with a Δβ greater than 0.1 that were unique to the tumour-normal pairs from EOCRC, 759 DMPs were hypermethylated, and 1777 DMPs were hypomethylated. (**c**) Barplot illustrating differences in methylation levels (Δβ) between tumour and normal pairs from EOCRC (blue), IOCRC (green), and LOCRC (yellow) for the top 20 DMPs ranked by statistical significance (genes that overlap DMPs are shown). (**d**) Volcano plots illustrating tumour-normal hypo- (<0) and hyper-methylated (>0) DMPs for EOCRC, IOCRC, and LOCRC. Log FC (of M-values) are shown on *x*-axis, whilst logged adjusted *p*-values are shown on *y*-axis. The red line denotes the *adj p* = 0.01 cut-off, and individual red dots represent statistically significant DMPs.

The pairwise analysis of matched CRC and NM samples within the three defined age groups identified differentially methylated probes (DMPs): 29% of all (431,942) CpGs in 41 EOCRCs, 34% in 34 IOCRCs, and 30% DMPs in 27 LOCRCs (Figure 1 and Table 2). These DMPs were associated with 17,666, 20,221, and 17,769 differentially methylated regions or genes (DMRs) in EOCRC, IOCRC and LOCRC groups, respectively. Hypomethylated DMRs represented 60%, 61%, and 53% of the overall DMRs in the EOCRC, IOCRC, and LOCRC groups, respectively (Table 2), and the difference in proportions was statistically significant (*p* < 0.001).

We identified 88,385 DMPs that overlapped between the three CRC-onset groups; however, each age group also showed a subset of unique DMPs (Table 2 and Figure 1a). Of the 18,755 DMPs that were unique to the EOCRC group, 39% were hypermethylated in CRCs when compared with matched NM samples. This proportion of hypermethylated CpGs was different from IOCRC and LOCRC (Table 2). When restricting to DMPs showing large changes in methylation (absolute mean Δβ values >0.1), the number of EOCRC DMPs was reduced to 2536 DMPs with 759 (30%) hypermethylated and 1777 (70%) hypomethylated CpGs in tumours (Figure 1b,c). A kernel smoothing of these 2536 CpGs revealed 234 DMRs (Supplementary Table S1). The top-ranked DMRs (by Stouffer’s *p*-value [34]) included: (1) the transcription start site (~2 kb) of the *TFAP2A* gene, (2) a short intergenic region on chromosome 1, and (3) a ~200 bp region located ~1 kb upstream of the *GSX1* gene. The proportions of CRCs with an aberrant DNAm greater than 0.1 (Δβ > 0.1) compared with matched normal, at each of the 234 EOCRC DMRs across each of the three age-of-onset groups are shown in Supplementary Table S1. In 158 of these 234 EOCRC DMRs, at least 50% of our 41 EOCRCs showed aberrant methylation (Δβ > 0.1), whereas it was less common in IOCRC and LOCRC groups (Supplementary Table S1). 

Restricting to DMRs with absolute mean differences in methylation (Δβ) > 0.1 to remove genes with subtle methylation changes which are less likely to impact gene function, we performed the KEGG analysis to examine biological pathways associated with DMRs for each age group. For the EOCRC group, 5585 DMRs (Supplementary Table S2) were associated with 12 KEGG pathways (FDR-adjusted *p* < 0.05) (Supplementary Figure S3 and Supplementary Table S3). For the IOCRC and LOCRC groups, 12 and 19 KEGG pathways (Supplementary Tables S4 and S5) were associated with 7701 and 7928 DMRs (Supplementary Tables S6 and S7), respectively. Ten KEGG pathways were common to all three age groups, including the “Neuroactive ligand-receptor interaction” pathway, which was the most significant pathway for all three onset groups. The “Maturity onset diabetes of the young” (MODY) pathway was uniquely associated with the DMRs from the EOCRC group. Of 26 total genes are associated with the MODY pathway, we found that 12 (46.2%) were differentially methylated in EOCRCs.

We next investigated the 234 EOCRC-associated DMRs in colon adenocarcinoma (COAD) and rectal adenocarcinoma (READ) tumours from the TCGA with HM450K data and relevant clinical information (e.g., age at diagnosis). There were 47 EOCRC (CRC Dx < 50 with mean Dx = 43.7 years ± 5 s.d.) and 164 LOCRC (Age Dx > 70 with mean Dx = 78.6 years ± 5.8 s.d.). Due to insufficient normal mucosa samples (*n* = 3) for EOCRC cases, the comparison between tumour and normal tissue samples was not possible; therefore, we examined the proportion of the 234 EOCRC-associated DMRs that were differentially methylated in the same direction as the 47 EOCRC when compared with the 164 LOCRC samples from the TCGA, controlled for gender. Of the 234 DMRs, 13 were significantly differentially methylated in the EOCRC TCGA tumours (Supplementary Table S1).

### 3.2. Methylomic Drift in Normal Colonic Mucosa and Colorectal Carcinogenesis

**Figure 2:**
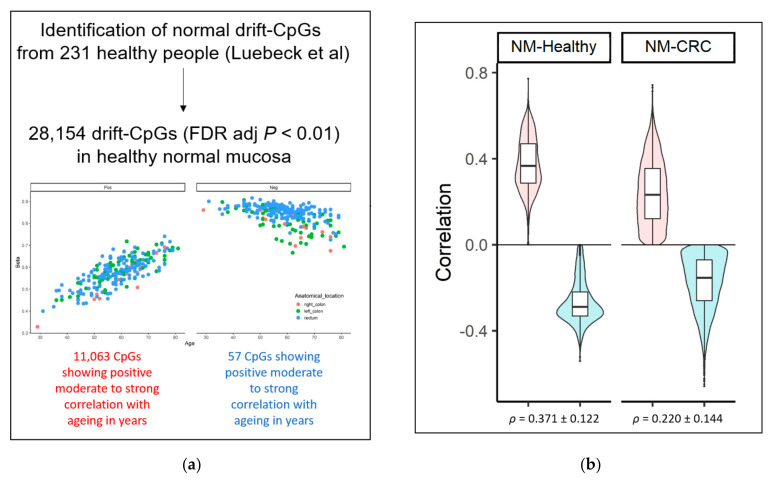

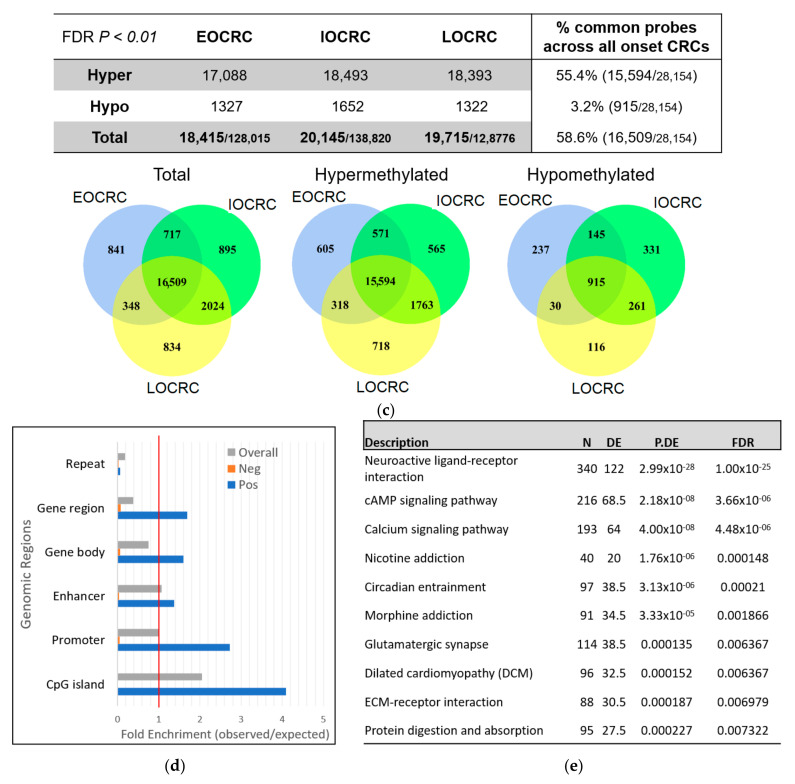
CpGs associated with the methylomic drift in normal colonic mucosa and those that are also DMPs in CRCs (**a**) 28,154 CpGs were identified where methylation levels were either positively or negatively correlated with increasing age, i.e., “drifts-CpGs”. (**b**) Spearman’s correlation coefficients for 26,886 positively correlated probes (red) and 1268 negatively correlated probes (blue) in normal mucosa from healthy control (“NM-healthy”, left) and people with CRC from this study (“NM-CRC”, right). (**c**) A table and Venn diagrams showing the breakdown of hypermethylated and hypomethylated cancer-associated drift-CpGs each for EOCRC, IOCRC, and LOCRC. (**d**) The representation of genomic regions (CpG island, gene promoter, enhancer, gene body, and repeat) for 16,509 cancer-associated drift CpGs common between all three age groups that were either positively or negatively correlated drift-CpGs. (**e**) Top 10 KEGG pathways associated with 16,509 cancer-associated drift CpGs.

We identified CpGs at which DNAm levels consistently change or “drift” with ageing using a previously published dataset of 231 NM samples collected from only people described as “healthy”, unaffected by CRC and ranging in age from 29 to 81 years (mean = 59 ± 10 s.d.) [14,37,38]. After adjusting for the anatomical site (proximal and distal colon and rectum), the analysis revealed 30,465 drift-CpGs (FDR adj *P* < 0.01) (Figure 2 and Supplementary Table S8). The majority (29,085 CpGs or 96%) were positively correlated (Spearman’s ranked correlated coefficient ρ > 0) with age (Figure 2). In the 129 NM samples from people with CRC, the association between methylation levels and age was weaker (Figure 2b) when tested on 28,154 drift-CpGs (excluding 2311 poorly performing probes which were filtered out in our data).

We next measured the overlap between the 28,154 drift-CpGs and the DMPs identified from our three CRC age groups. The majority of these drift-CpGs were aberrantly methylated in tumours, with 65%, 72%, and 70% of the 28,154 drift-CpGs overlapping with the DMPs from the EOCRC, IOCRC, and LOCRC groups, respectively (Figure 2c). Intriguingly, these cancer-associated drift CpGs (“CA-drift CpGs”) were broadly affected even in EOCRC, with 59% (16,509/28,154) of them common across all three age groups. The CA-drift CpGs clustered into 2824 DMRs linked to 2597 annotated genes and 227 intergenic regions (Supplementary Table S9).

Almost all 15,982 CA-drift CpGs, for which methylation was positively correlated with ageing, were hypermethylated (15,578 CpGs or 97%) in tumours, and this pattern was consistent across the three age-of-diagnosis groups. The CA-drift CpGs were located largely within intragenic and regulatory regions, including gene promoter and enhancer regions, while CpGs within repeat and gene body regions were underrepresented (Figure 2d). A KEGG pathway analysis of the 16,509 CA-drift CpGs identified several biological pathways associated with genes overlapping those CpG sites, as shown in Figure 2e.

**Figure 3:**
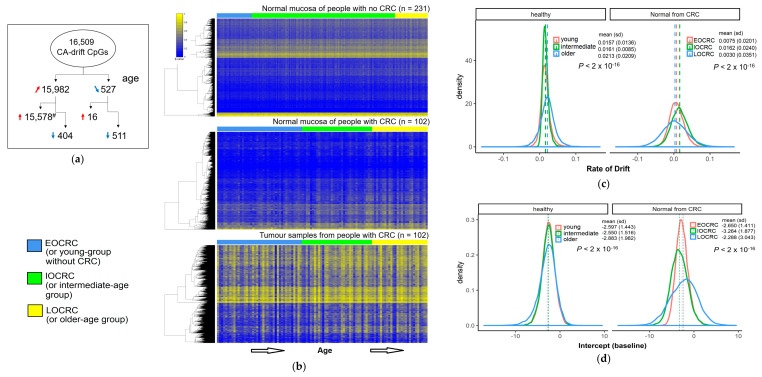
(**a**) Schema describing number breakdowns for CA-drift CpGs positively (red arrow) and negatively (blue arrow) correlated with increasing age and hyper (red) and hypomethylated (blue) in CRCs. ^#^ CpGs included in subsequent analyses (**b**) DNA methylation heatmap illustrating methylation patterns across 15,578 CA-drift CpGs (*y*-axis) for 231 normal mucosa samples (*x*-axis) from the healthy group. Individual samples are shown along the horizontal axis and order by increasing age for 102 normal mucosa samples and 102 tumour samples from people with CRC. (**c**) Density plots showing the distribution of the drift rate for young/early (or EOCRC), intermediate (or IOCRC), and late age (or LOCRC) group samples from people without CRC and with CRC. (**d**) Density plots showing the baseline of the drift rate (using intercept as surrogate).

Of the 28,154 drift-CpGs identified, 3% (841/28,154 CpGs) overlapped with cancer DMPs that were unique to EOCRC (Figure 3a), which clustered into 86 regions (80 annotated genes and 6 intergenic) (Supplementary Table S10). The *GSX1* and *TFAP2A* regions mentioned in the above analysis were among the highly ranked CA-drift CpGs specific to EOCRC. Other genes associated with the CA-drift CpGs of EOCRC (Supplementary Table S10) included *PRRT1* (gene body region), *PHF11* (intronic region), and *ZFP42* (transcription start site and 5’UTR). We observed enrichment of the “Systemic lupus erythematosus” (FDR-adj. *p* = 0.0002) and “Alcoholism” (FDR-adj *p* = 0.004) KEGG pathways associated with the 86 regions.

### 3.3. Rate of Change of DNA Methylation at Cancer-Associated Drift CpGs

The rate of DNAm changes (or drift) at the CA-drift CpGs in NMs from people without CRC (mean changes in β/10yrs = 0.02 ± 0.010 s.d.) and people with CRC (mean changes in β/10yrs = 0.007 ± 0.014 s.d.) were significant different (*p* < 0.001), although both groups show slow rates. In contrast, a substantial degree of DNAm changes at the CA-drift CpGs were observed in CRC tumour samples irrespective of the age of diagnosis (Figure 3a,b). We evaluated the drift rate at individual 15,578 CpGs that were hypermethylated and positively correlated with increasing age by fitting a linear model between the age and M-values for individual CA-drift CpGs as previously described [14].

Small but statistically significant differences in the pattern of the drift rates between the three age-of-diagnosis groups were observed for NMs from both healthy and CRC-affected groups (*p* < 0.001, Figure 3c). While the differences in mean drift rates did not show strong differences between the three age groups, the variations were notably large (*p* < 0.001) in the older age groups in NMs from both healthy and CRC-affected groups (Figure 3d). This trend between the three age groups was also significantly different between NMs from people with or without CRC (*p* < 0.001) due to larger variations in the drift rate for the CRC group overall. We also estimated the baseline of the drift for individual groups using the intercept from the linear model as a surrogate. Similarly, we observed small but significant differences between the three age groups but not between groups with CRC and without CRC.

### 3.4. DNA Methylation-Based Biological Age of the Normal Mucosa and Age Acceleration

We assessed the “epigenetic age” of each tissue sample using five well-established DNAm-based clock algorithms [16,17,19], including two recently developed “mitotic-like” clocks, epiTOC [21], and epiTOC2 [20]. These two mitotic-like DNAm clocks have demonstrated their effectiveness in discriminating precancerous from normal tissues, showing utility for predicting cancer risks from pre-diagnostic tissue samples [20,21]. Unlike non-mitotic DNAm clocks, these clocks quantify the magnitude of DNAm errors that arose throughout every stem cell division by interrogating DNAm levels across the promoters of polycomb-associated genes [20,21].

**Figure 4:**
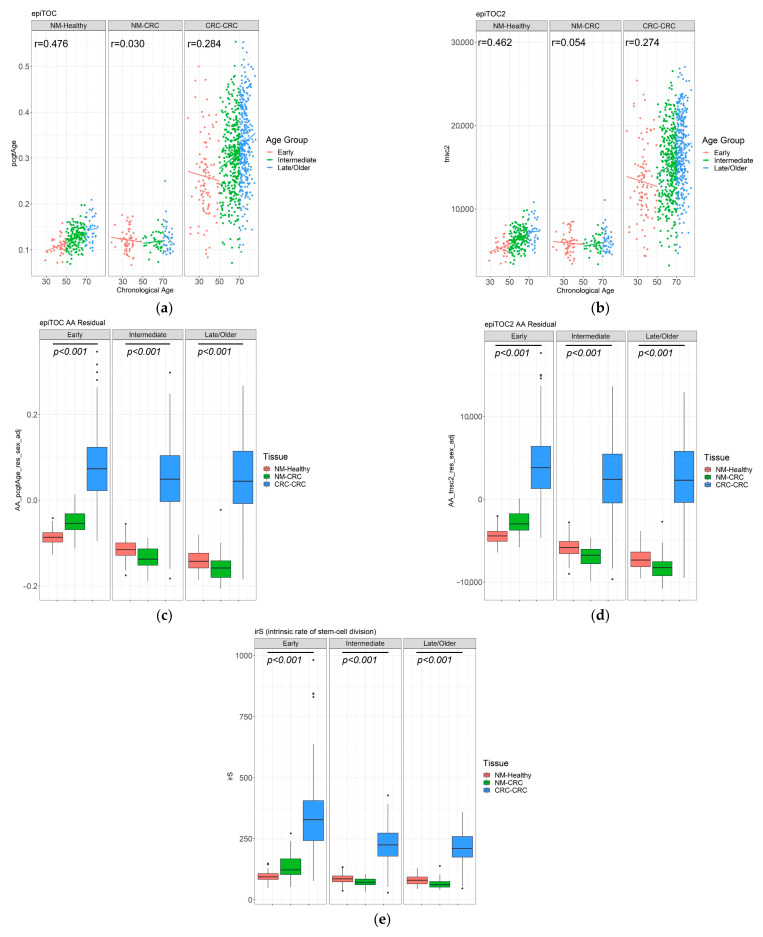
The mitotic-based DNAms age prediction by epiTOC and epiTOC2 for normal colonic mucosa samples from people without (“NM-Healthy”) and people with CRC (“NM-CRC”), and CRC tumour (“CRC-CRC”) samples, separated into Early, Intermediate and Late/Older age groups based on the age at tissue collection or CRC diagnosis. (**a**) Scatterplots illustrating correlations between chronological age (*x*-axis) and epiTOC DNAm-based age (*y*-axis) for corresponding samples. (**b**) Scatterplots illustrating correlation between chronological age and epiTOC2 DNAm-based age (*y*-axis). Boxplots illustrating the distribution of age-acceleration (AA) as estimated using epiTOC (**c**), epiTOC2 (**d**), and irS (average lifetime intrinsic rate of stem-cell division per sample), as derived from epiTOC2 (**e**), shown separately by the age and the tissue groups. *P*-values were obtained from Kruskal-Wallis Rank Sum tests.

By both epiTOC and epiTOC2, moderate correlations (r = 0.462 and 0.476, respectively) were observed between DNAm age and chronological age in NMs from people without CRC, whereas this was notably poorer in NMs from people with CRC (r = 0.030 and 0.054, respectively) (Figure 4a,b). In CRCs, weak correlations (r = 0.284 and 0.274 for epiTOC and epiTOC2, respectively) were observed but with a greater and broader range of DNAm ages compared to the two NM groups. All three non-mitotic clocks (i.e., Horvath [17], Hannum [16], and PhenoAge [19]) generally showed moderate-to-strong correlations in NMs, as previously demonstrated [43] but not in CRCs (Supplementary Figure S4).

Using the five epigenetic clocks, we assessed AA by estimating residuals by regressing DNAm (or mitotic) ages onto the chronological ages for all samples [42,43] and adjusting for sex. Additionally, the average intrinsic rate of stem-cell division (irS) per year was also derived from the epiTOC2 algorithm [20]. In all age groups, CRCs showed consistent and statistically significant AA by epiTOC (*p* < 2 × 10^−16^), epiTOC2 (*p* < 2 × 10^−16^) (Figure 4c,d), and also by irS (*p* < 2 × 10^−16^). The same pattern was also observed in the Hannum clock and PhenoAge, but not in the Horvath clock (Supplementary Figure S4).

To further exploit both mitotic clocks’ efficacy for estimating the cancer risk in non-cancerous normal tissues, we next applied both epiTOC and epiTOC2 to NM samples, with the primary aim to address whether AA or the intrinsic stem cell division rates differ between NMs from people with and without CRC, across the age groups. To eliminate the likely skew caused by the substantial AA in CRCs, we performed a linear regression by only including 129 and 231 NMs from people with and without CRC, respectively. This analysis was further adjusted for sex, as well as cell heterogeneities, by incorporating the ratios of epithelial, fibroblast, and immune cell compositions, which were estimated using the EpiDISH [44].

**Figure 5:**
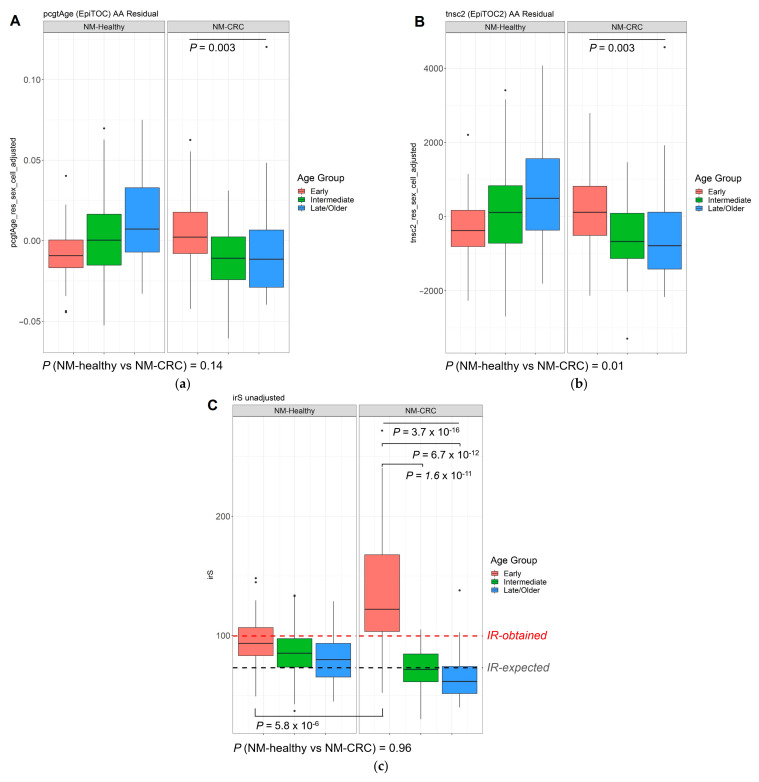
Boxplots showing the distributions of AA measured by epiTOC (**a**), epiTOC2 (**b**), and irS (**c**), in NMs from people without (“NM-Healthy”) and people with CRC (“NM-CRC”). AA was measured by calculating residuals from a linear regression analysis of DNAm age on chronological age for each individual. *p*-values between two tissue types (NM-Healthy vs. NM-CRC) were obtained by performing Wilcox tests, whereas *p*-values between three age groups were obtained from Kruskal-Willis tests. In (**c**), the red horizontal line denotes previously computed average irS (IR) in normal colonic mucosa [20], and the black horizontal line denotes an expected experimentally derived stem-cell division rate for colorectal tissue [21,45].

Both epiTOC and epiTOC2 predicted significant AA in NMs from EOCRC compared with NMs from IOCRC and LOCRC (*p* = 0.003 for both epiTOC and epiTOC2; Figure 5). In NMs from healthy controls without CRCs, a reverse pattern was observed by both clocks (Figure 5a,b), although this skew was likely induced by obtaining residuals from the linear regression due to the skewed coefficients between two NM groups.

The irS was predicted to be significantly elevated in NM samples from the EOCRC group (*p* = 3.7 × 10^−16^, Figure 5c). The epiTOC2 clock measures the average lifetime intrinsic rate of stem-cell division per year for each sample, where this has been demonstrated to distinguish precancerous normal colonic lesions [20] accurately. The irS, estimated by the epiTOC2 (and automatically adjusted for age), ranged from 37 (indicative of an average 37 stem cell turnover per year) to 48 (mean = 86.7 ± 20 SD) for 231 NMs from healthy people, whereas for 129 NMs from people with CRCs it ranged from 30 to 271 (mean = 96.4 ± 46 SD; *p* = 0.96). A significantly higher irS was detected in the NMs from the EOCRC group when compared with the IOCRC (*p* = 1.6 × 10^−11^) and LOCRC (*p* = 6.7 × 10^−12^) groups, indicating a higher average stem-cell division rate in NMs from people with EOCRC (Figure 5c). Only the NM samples from the EOCRC group showed an overall irS (mean = 135, median = 122) that was higher than previously observed in epiTOC2 [20] or biologically-determined stem-cell division rate [45] in normal colonic tissue. No significant differences in AA between the three age groups were observed in the analyses of the three non-mitotic clocks (Supplementary Figure S4)

## 4. Discussion

In this study, we examined the genome-wide DNAm changes associated with colorectal tumourigenesis across the full spectrum of age. We reported 88,358 consensus DMPs (20% of total CpGs tested) between the three CRC age groups, representing key somatic DNAm-related changes in tumourigenesis. Beyond these consensus DMPs, we identified 18,775 unique DMPs for EOCRC, of which 2536 showed Δβ>0.1 and clustered into 234 DMRs, including the top-ranked DMRs related to the *TFAP2A* and *GSX1* genes. These genes have previously been reported to play a role in CRC tumourigenesis [46,47,48]. Of note, *GSX1* methylation levels have been linked to obesity-associated CRC [46]. Obesity is a recognised risk factor for EOCRC [49], which may support the hypothesis that risk factors such as obesity may predispose to EOCRC by modulating the DNAm of genes like *GSX1*. Of our 234 EOCRC DMRs, we found that 13 were also differentially methylated between 47 EOCRC and 164 LOCRC samples from TCGA, albeit there was a notable difference in mean diagnosis ages between the EOCRCs from the two cohorts. These 13 EOCRC-associated DMRs that were common to two cohorts may represent a robust biomarker in EOCRC. In addition, we found that the EOCRC DMRs were associated with the KEGG pathway “Maturity onset diabetes of the young”. Our findings may support the presence of unique DNAm molecular drivers and disrupted pathways in EOCRC.

Diabetes mellitus (DM) is a known risk factor for CRC [50]. Although the rising trend of DM has been proposed to contribute to an increasing incidence of EOCRC [51], no particular association between DM and EOCRC has been shown, although only a limited number of reports are available [9,52]. Maturity onset diabetes of the young (MODY) is a rare hereditary autoimmune condition that affects adolescents or young adults [53]. Like other forms of diabetes, MODY is linked to obesity and hyperglycaemia [53], which are known causative factors for intestinal inflammation and are also linked to gut microbiome dysbiosis [54]. In our EOCRC tumours, we found that approximately half of the genes associated with the MODY pathway were aberrantly methylated in EOCRC. While no information about this atypical form of diabetes was collected in our cohorts, MODY-related genes have been reported to be epigenetically dysregulated in CRCs (of no particular onset/subtype) [55] and hypermethylated with ageing in people at high risk for metabolic syndromes [56], warranting further investigation as a specific risk factor for EOCRC. Those 12 aberrantly methylated genes in the MODY pathway may need to be further validated for their functional implication in the future.

The adverse role of ageing-associated methylomic drift in predisposing people to CRC has become clearer and is now postulated to be the primary mechanism for explaining ageing-related CRC risk [13,14,57]. Recent data utilising the high-density methylation arrays have specifically predicted a long sojourn time from the early mostly benign ageing-associated methylation changes to the formation of premalignant cells and suggested that early methylation changes can be traced back several decades [14]. We hypothesised that EOCRC shows an accelerated ageing process with a more rapid transition time from benign to malignant cells and that this would be reflected in an increase in cancer-associated drift CpGs in EOCRC and/or by measures of AA in the NM of people with EOCRC. The absence of longitudinal sampling in our NM data meant we were unable to directly measure methylomic drift rates within each individual with EOCRC. However, we showed that a high proportion (>58%) of CpGs associated with methylomic drift are aberrantly methylated in CRC tumours of all ages. Remarkably, DNAm aberrations at these cancer-associated drift-CpGs were also widely affected in EOCRC, and this was as extensive as in IOCRC and LOCRC, which suggests that the DNAm of cancer-associated drift CpGs is essential for CRC tumourigenesis at all ages. Our data support the concept that EOCRCs more rapidly acquire extensive DNAm aberration at these drift-CpGs without needing a life-long accumulation of cancer-related methylomic drift at these drift CpGs. 

Cancer (and especially cancer of non-hereditary origin) is a phenomenon related to cumulative DNAm aberrations, including those of ageing-associated methylomic drift [13]. Methylomic drift, defined as a “gradual change away from baseline (i.e., birth or conception)”, is postulated to arise due to the imperfect epigenetic maintenance machinery [13]. Though the DNAm changes associated with methylomic drift are estimated to be subtle, tissues that are highly proliferative and directly exposed to exogenous factors (e.g., diet, microbiota) such as the colonic epithelium may have a faster drift than other tissues [13,58]. We hypothesise that, in our EOCRC tumours, defective epigenetic machinery or adverse environmental exposures may have contributed to this rapid transformation in relatively shorter periods of time. Further studies identifying the causative factors and predictive biomarkers for early detection may help prevent sporadic EOCRCs. A longitudinal sampling of NMs from the same individuals at a higher risk of CRC could be used to estimate the “endogenous” methylomic drift rate for each individual and tease out the effect of specific genetic factors and environmental exposures to this cancer-associated drift.

In NMs from people with EOCRC, we demonstrated significant AA, compared with NMs from people with IOCRC or LOCRC, or NMs from young, healthy people without CRC, as measured using mitotic-based DNAm clocks. By utilising two recently developed mitotic-based DNAm clocks, we showed that the NMs from the EOCRC group exhibit predicted total mitotic stem cell division numbers that were comparable to the NMs of IOCRC and LOCRC groups, suggesting an elevated mitotic rate per given years of age in our EOCRC group. The irS tool estimates the average intrinsic rate of stem cell division per year. We found further support that accelerated ageing in NMs from people with EOCRC, evidenced by the only group showing the mean irS exceeding the expected stem-cell division rates in healthy colonic mucosa (37,55) and significantly different from the NMs of IOCRC, LOCRC, or the NMs of people without CRC.

The increased mitotic rate and DNAm errors that arise from this have been proposed to be a major determinant of cancer risk [13,20,21,59,60], and here, we have shown accelerated ageing in non-cancerous NMs from people who developed EOCRC, which may constitute an important risk predictor for EOCRC development. The epiTOC and epiTOC2 mitotic-based clocks used in this study have been proven to accurately predict an increase in cell proliferation, a phenomenon associated with inflammatory and precancerous tissues, various cancer types, and even in the buccal epithelial cells of smokers [20,21], demonstrating its potential as a cancer-risk estimator in pre-diagnostic tissue samples. It is likely that the mitotic rate of individual tissue results from an interplay between intrinsic and extrinsic factors such as inflammation, environmental, and the gut microbiome [13,60], and we hypothesise that disruption of these factors may be reflected in the accelerated (mitotic) age in NMs from EOCRC. While this hypothesis remains plausible, our study was not designed to directly assess such association. Future studies investigating this potential link between specific intrinsic and extrinsic factors and disruptions in the mitotic rate could have particular importance for EOCRC prevention.

CpG island methylator phenotype (CIMP)-high or CIMP-positive CRC represents ~20% of CRC and is associated with the serrated pathway of tumourigenesis and an older age at diagnosis [61]. This subtype of CRC is characterised by widespread and concordant DNA methylation changes [61]. In our study, CIMP-positive CRCs represented only a small proportion of all CRCs (we excluded CRCs identified by *MLH1* promoter methylation and loss of MLH1/PMS2 protein expression (MMR-deficient)). In addition, the CA-drift CpGs showed minimal overlap with commonly assessed CIMP-related CpGs; therefore, CIMP is unlikely to be the underlying driver of the CA-drift CpGs reported in this study. 

This study has some limitations. We utilised a public dataset of “healthy” NM samples; therefore, some inherent technical variations (e.g., batch effect, tissue source) were unavoidable. A possible disparity due to tissue source (fresh-frozen vs. FFPE-derived DNA) or batch effect cannot be ruled out. To partly address this, we have not performed any direct comparisons (e.g., differential methylation) between the public healthy NM data and our NM or CRC data. The public NM samples were only compared amongst other samples in the same group to identify CpGs associated with methylation drift. Ideally, a set of colonic NM samples collected longitudinally from the same individuals would serve as a perfect archetype to investigate this though we are not aware of such a cohort. Another limitation is the lack of gene expression data to assess the functional implications of the methylation changes. Although we have used KEGG pathways to explore the common ontologies related to our DMRs, genes related to the mentioned KEGG pathways may require further examination for their subsequent functional (i.e., gene expression) implications.

## 5. Conclusions

Our study represents one of the largest genome-wide DNAm studies on EOCRC. Utilising participants recruited via two well-characterised cohorts, we have generated genome-wide DNAm data for 769 non-hereditary, MMR-proficient CRCs, including 97 tumour samples (with 41 matched tumour-normal pairs) from people with EOCRC. Several novel findings from this study include: (i) extensive DNAm alterations in all CRCs including EOCRCs, indicative of the essential role DNAm plays in CRC development at all ages; (ii) the identification of DNAm-related changes specific to EOCRC including at the *TFAP2A* and *GSX1* genes, and 12 differentially methylated genes associated with the MODY pathway; (iii) EOCRC tumours have extensive DNAm aberrations at ageing-associated CpGs or CA-drift CpGs without requiring the decades-long accumulation of methylation aberrations, which has been postulated to be essential in conventional CRC development that commonly occurs at an older age [13,14]; and (iv) AA as measured by epiTOC2, which is an epigenetic clock for predicting mitotic stem cell division rates (i.e., accelerated tissue ageing), in NMs from people with EOCRC. These findings suggest that tumourigenesis in EOCRC involves widespread and distinct DNAm alterations, including those commonly associated with the ageing process of colonic epithelial cells. Further studies investigating the cause of these DNAm changes will help elucidate the determinants of the increasing incidence of EOCRC and aid in the identification of predictive biomarkers such as accelerated epigenetic ageing that can lead to new prevention strategies in young people who have an elevated risk of EOCRC.

## Figures and Tables

**Table 1:** CRC-affected participant and tumour characteristics for early, intermediate, and late-onset groups.

	Onset of Disease	Early (EOCRC)		Intermediate (IOCRC)		Late (LOCRC)	
	Ages of onset	<50 years		≥50 and <70 years		≥70 years	
	Mean (sd)	36.2 (6.2) years		62.6 (5.2) years		74.8 (3.5) years	
	Total number of cases (matched tumour/normal pairs)	110 (41)		334 (34)		325 (27)	
	Sex (%)	F	M	F	M	F	M
		64 (58)	46 (42)	158 (47)	176 (53)	142 (43)	183 (57)
	Tissue type	Tumour	Normal	Tumour	Normal	Tumour	Normal
		97	54	343	35	318	40
Anatomical site (%)	Right colon	20 (20.5)	11 (21)	86 (25)	8 (23)	107 (34)	14 (35)
	Left colon	32 (33.5)	13 (24)	100 (29)	8 (23)	91 (29)	9 (22.5)
	Rectum	40 (41)	19 (35)	138 (40)	8 (23)	114 (36)	7 (17.5)
	unknown	5 (5)	11 (20)	19 (6)	11 (31)	6 (2)	10 (25)
TNM (AJCC) stage (%)	Stage I	17 (18)	-	70 (20)	-	75 (24)	-
	Stage II	25 (26)	-	81 (24)	-	81 (25)	-
	Stage III	43 (44)	-	101 (30)	-	87 (27)	-
	Stage IV	9 (9)	-	54 (16)	-	55 (17)	-
	unknown	3	-	37	-	20	-
CIMP group	CIMP +	0	-	19	-	34	-
	CIMP -	93	-	320	-	280	-
	unknown	4	-	4	-	4	-

**Table 2:** Numbers of differentially methylated probes (DMPs) and regions (DMRs) identified between tumour and normal pairs from EOCRC, IOCRC, and LOCRC cases included in the study.

		Early-Onset (EOCRC)	Intermediate-Onset (IOCRC)	Late-Onset (LOCRC)	*p*-Value
**Age of Dx**		<50 years	≥50 and <70 years	≥70 years	
N of tumour and matched normal pairs		41	34	27	
Number of DMPs *	Hypermethylated	49,044 (38.8%)	59,125 (40.3%)	57,970 (45.1%)	<0.001 ^#^
	Hypomethylated	77,228 (61.2%)	87,448 (59.7%)	70,806 (54.9%)	
	Total	126,272 (29.2% of all CpGs included in the analysis)	146,573 (33.9%)	128,776 (29.8%)	
Number of DMRs	Hypermethylated	7102 (40%)	7985 (39.5%)	8407 (47.3%)	<0.001 ^#^
	Hypomethylated	10,564 (60%)	12,236 (60.5%)	9362 (52.7%)	
	Total	17,666	20,221	17,769	
Number of DMPs * that are only presented in each group	Hypermethylated	7369 (39.3%)	9171 (40%)	10,216 (69%)	<0.001 ^#^
	Hypomethylated	11,386 (60.7%)	13,727 (60%)	4581 (31%)	
	Total	18,755 (4.3%)	22,898 (5.3%)	14,797 (3.4%)	

^#^ Pearson’s Chi-squared test. * FDR adj. *P* < 0.01

## Data Availability

The datasets used in this current study are available from the corresponding author on reasonable request and subject to ethics approval.

## Supplementary Materials

The following are available online at https://www.mdpi.com/article/10.3390/cancers13112589/s1, Supplementary Figure S1 A flow diagram showing the data processing steps for deriving DNAm-age from 231 NMs (without CRC), 129 NMs from people with CRC, and 758 CRC tumour samples. (a) as described in Teschendorff 2020, (b) using the online calculator (http://dnamage.genetics.ucla.edu/new), and (c) using the ENmix Bioconductor package. Supplementary Figure S2 PCA plot (A) and β methylation density plot (B) showing overall DNA methylation similarities between samples. Individual samples are coloured by tissue type (tumour/normal mucosa). PCA plots for tumour (C) and normal colonic mucosa samples only (D). Supplementary Figure S3 KEGG pathways associated with differentially methylated genes of EOCRC, IOCRC, and LOCRC. Numbers of KEGG pathways unique to each CRC onset group as well as numbers of overlapping pathways are shown. Supplementary Figure S4 DNA methylation age and AA measured using non-mitotic epigenetic clocks. Scatterplots showing the correlations between DNAm-derived ages using the Horvath (A) and Hannum (B) clocks, and PhenoAge (C) for 231 NMs from healthy people (“NM-Healthy”), 129 NMs from people with CRC (“NM-CRC”) and 758 CRC tumour samples (“CRC-CRC”). Age Acceleration (AA) was estimated by obtaining residuals from a linear regression of DNAm-ages on chronological age and adjusting for sex. Boxplots showing the AA distributions, as estimated by the Horvath (D) and Hannum (E) clocks, and PhenoAge (F). *P*-values were computed by Wilcoxon Rank Sum test for two groups (NM-Healthy vs. NM-CRC) or by Kruskal-Wallis Rank Sum test for three groups (NM-Healthy vs. NM-CRC vs. CRC-CRC). Table S1: A list of differentially methylated regions (DMRs) uniquely associated with EOCRC. Table S2: All DMRs associated with EOCRC showing absolute mean differences (between tumour and normal mucosa) of >0.1. Table S3: KEGG pathways associated with differentially methylated genes between tumour and normal mucosa from EOCRC. Table S4: KEGG pathways associated with differentially methylated genes between tumour and normal mucosa from intermediate-onset CRC. Table S5: KEGG pathways associated with differentially methylated genes between tumour and normal mucosa from late-onset CRC. Table S6: All DMRs associated with IOCRC showing absolute mean differences (between tumour and normal mucosa) of >0.1. Table S7: DMRs associated with LOCRC showing absolute mean differences (between tumour and normal mucosa) of >0.1. Table S8: List of CpGs associated with ageing in 231 normal colonic mucosa samples from healthy people without CRC. Table S9: 2824 DMRs associated with CA-drift CpGs. Table S10: DMRs associated with CA-drift-CpGs specific to EOCRC.
