# Supplementary material for: DNA Methylation Signatures and the Contribution of Age-Associated Methylomic Drift to Carcinogenesis in Early-Onset Colorectal Cancer
Source: Cancers (Basel). 2021 May 25;13(11):2589. doi: 10.3390/cancers13112589 (PMC8199056; doi:10.3390/cancers13112589)
Supplement: Supplementary file 1 [file cancers-13-02589-s001.zip › cancers-1205235-supplementary-for xml/cancers-1205235-supplementary-for xml.pdf]

cancers

MDPI

Supplementary Materials: DNA Methylation Signatures and the Contribution of Age-Associated Methylomic Drift to Carcinogenesis in Early-Onset Colorectal Cancer

Jihoon E. Joo, Mark Clendenning, Ee Ming Wong, Christophe Rosty, Khalid Mahmood, Peter Georgeson, Ingrid M. Winship, Susan G. Preston, Aung Ko Win, Pierre-Antoine Dugué, Harindra Jayasekara, Dallas English, Finlay A. Macrae, John L. Hopper, Mark A. Jenkins, Roger L. Milne, Graham G. Giles, Melissa C. Southey and Daniel D. Buchanan

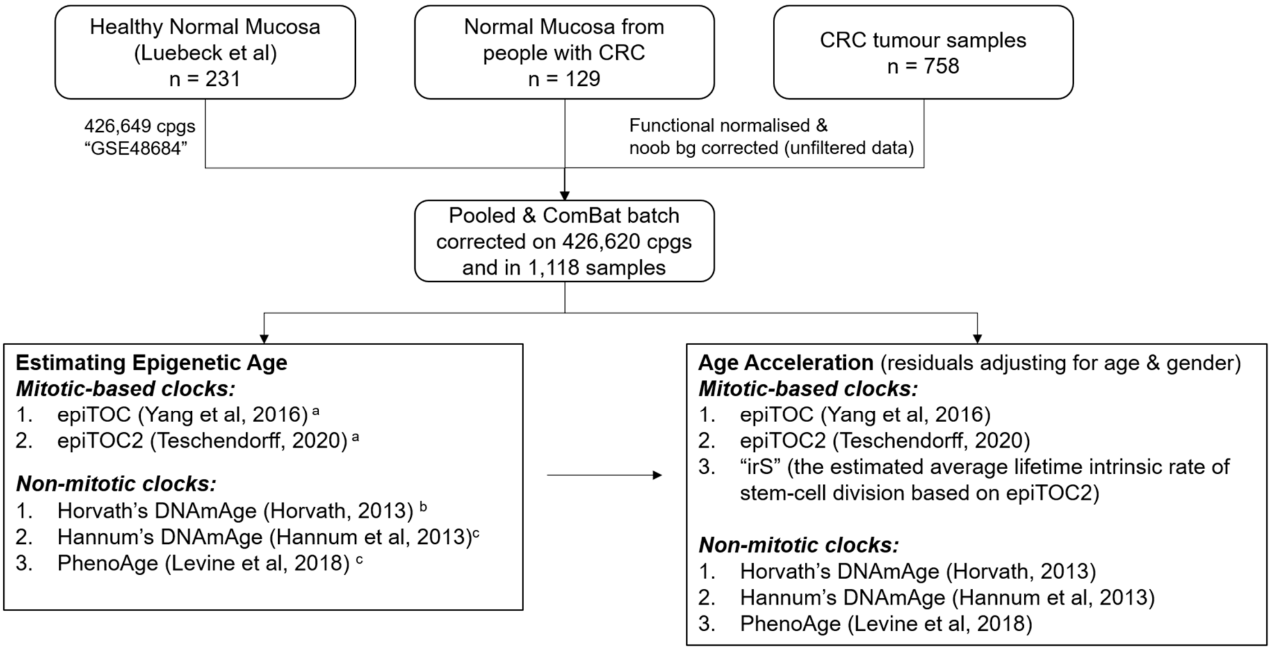

Figure S1. A flow diagram showing the data processing steps for deriving DNAm-age from 231 NMs (without CRC), 129 NMs from people with CRC, and 758 CRC tumour samples. (a) as described in Teschendorff 2020, (b) using the online calculator (http://dnamage.genetics.ucla.edu/new), and (c) using the ENmix Bioconductor package.

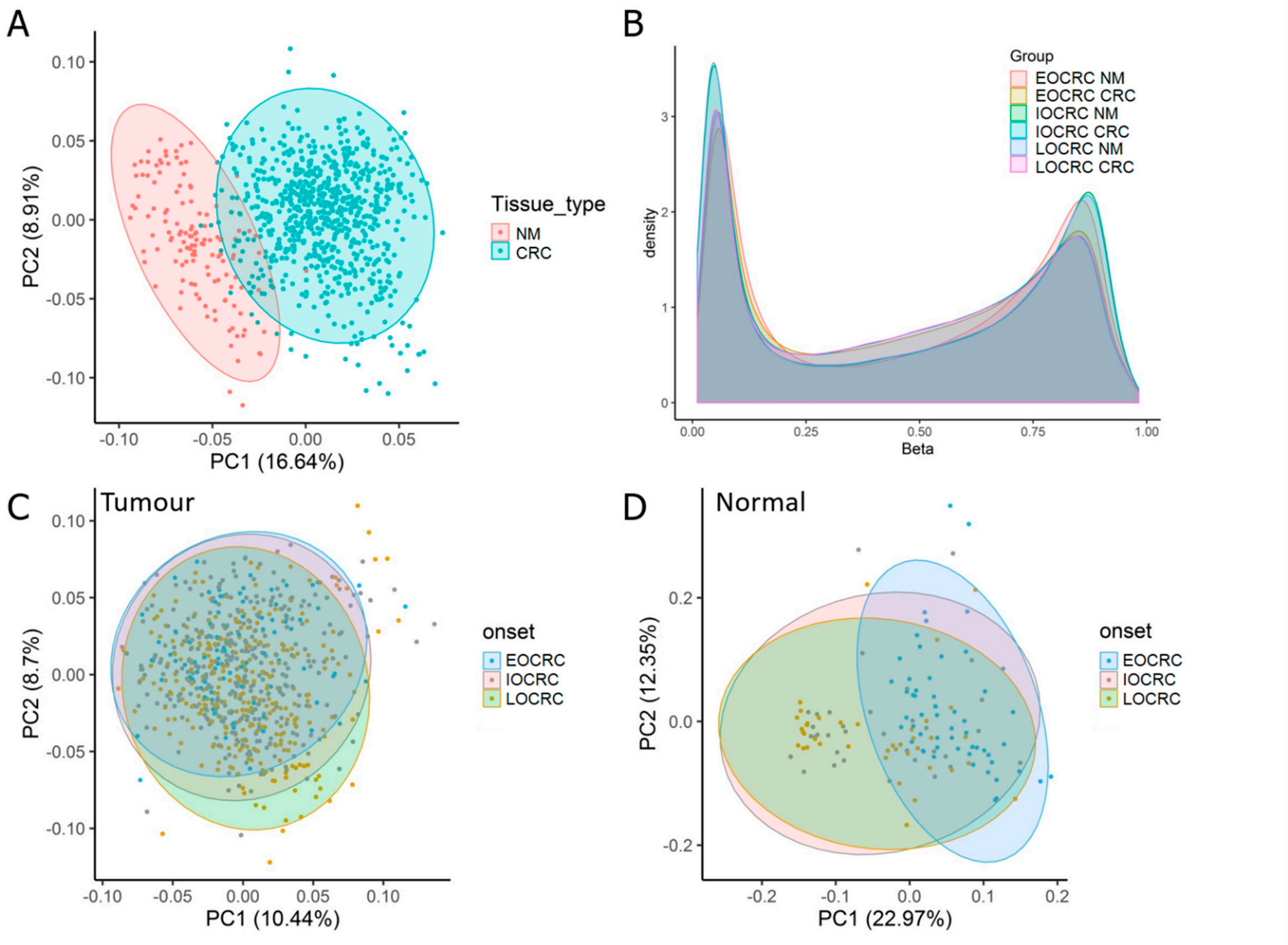

Figure S2. PCA plot (**A**) and β methylation density plot (**B**) showing overall DNA methylation similarities between samples. Individual samples are coloured by tissue type (tumour/normal mucosa). PCA plots for tumour (**C**) and normal colonic mucosa samples only (**D**).

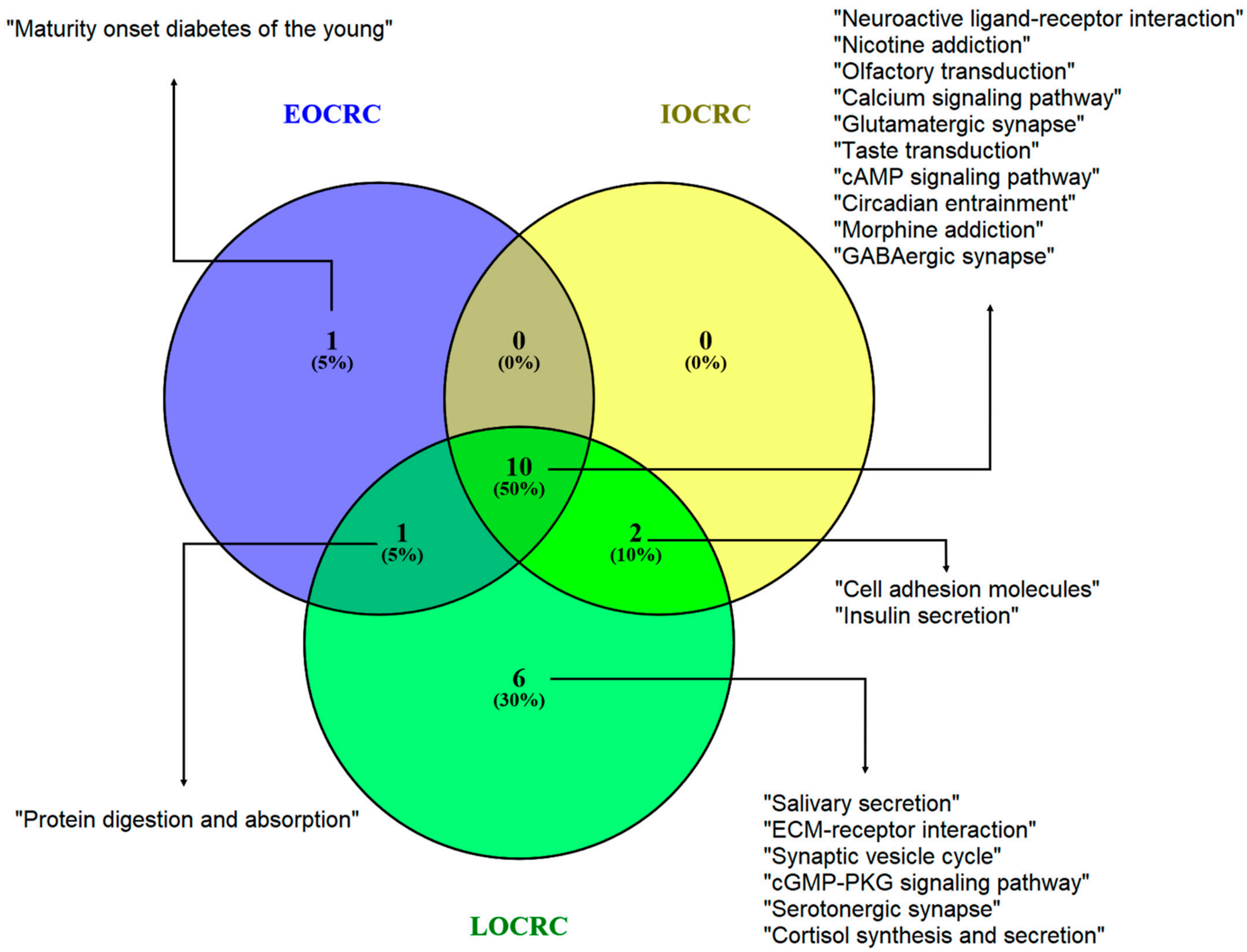

Figure S3. KEGG pathways associated with differentially methylated genes of EOCRC, IOCRC, and LOCRC. Numbers of KEGG pathways unique to each CRC onset group as well as numbers of overlapping pathways are shown.

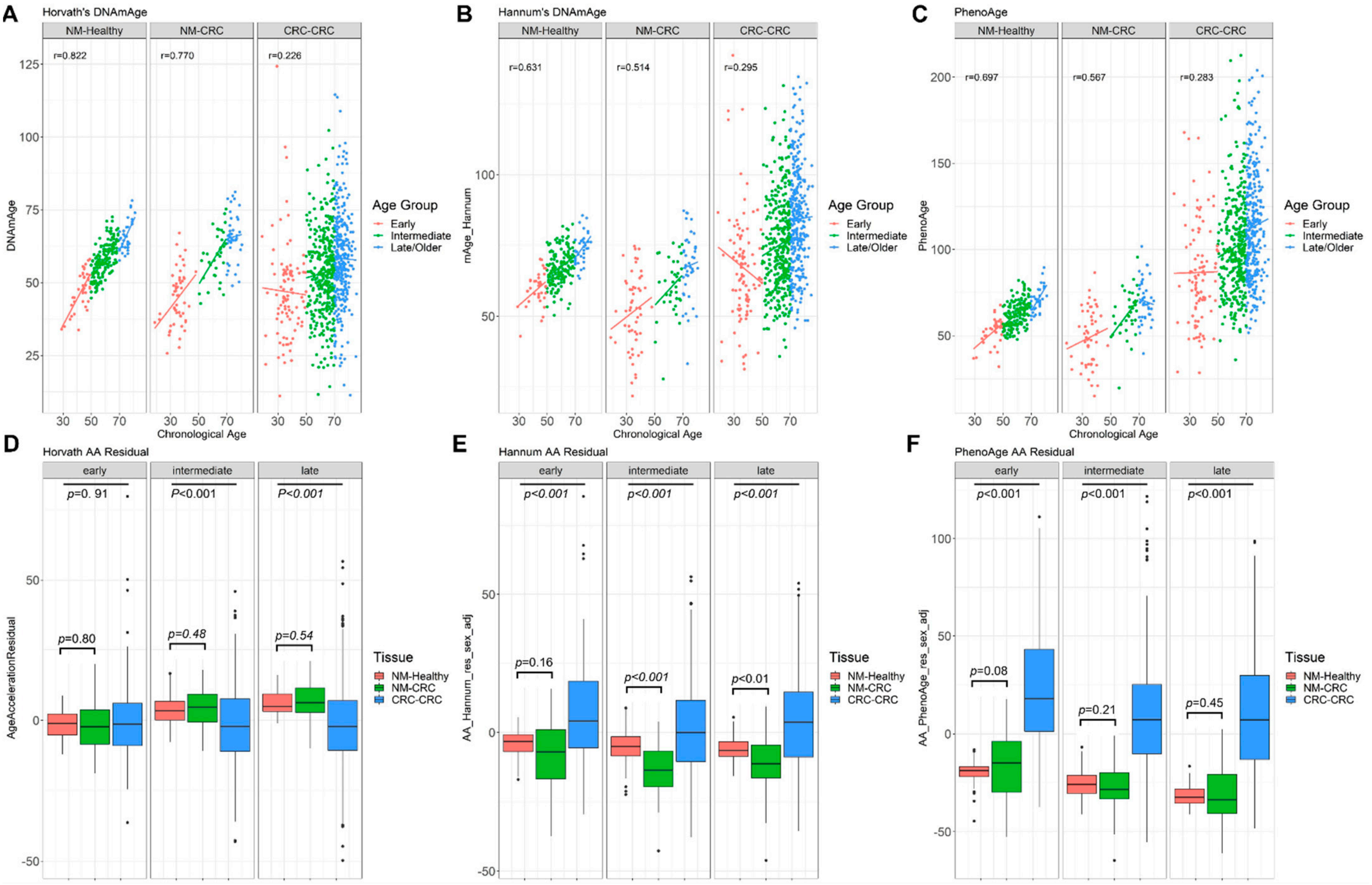

Figure S4. DNA methylation age and AA measured using non-mitotic epigenetic clocks. Scatterplots showing the correlations between DNAm-derived ages using the Horvath (**A**) and Hannum (**B**) clocks, and PhenoAge (**C**) for 231 NMs from healthy people ("NM-Healthy"), 129 NMs from people with CRC ("NM-CRC") and 758 CRC tumour samples ("CRC-CRC"). Age Acceleration (AA) was estimated by obtaining residuals from a linear regression of DNAm-ages on chronological age and adjusting for sex. Boxplots showing the AA distributions, as estimated by the Horvath (**D**) and Hannum (**E**) clocks, and PhenoAge (**F**). *P*-values were computed by Wilcoxon Rank Sum test for two groups (NM-Healthy vs. NM-CRC) or by Kruskal-Wallis Rank Sum test for three groups (NM-Healthy vs. NM-CRC vs. CRC-CRC).
